# Physical, behavioral, and hormonal changes in the resumption of sexual receptivity during postpartum infertility in female bonobos at Wamba

**DOI:** 10.1007/s10329-021-00968-w

**Published:** 2022-02-10

**Authors:** Chie Hashimoto, Heungjin Ryu, Keiko Mouri, Keiko Shimizu, Tetsuya Sakamaki, Takeshi Furuichi

**Affiliations:** 1 Primate Research Institute, Kyoto University, Kyoto, Japan; 2 School of Life Science, Ulsan National Institute of Science and Technology, Ulsan, Republic of Korea; 3 Faculty of Science, Okayama University of Science, Okayama, Japan; 4 Antwerp Zoo Foundation, Royal Zoological Society of Antwerp, Antwerp, Belgium

**Keywords:** Bonobo, *Pan paniscus*, Postpartum infertility, Operational sex ratio, Sexual swelling, Copulation, E_1_C concentration

## Abstract

The operational sex ratio (OSR) is used as a predictor for the intensity of mating competition. While many factors affect the OSR, there tends to be a high male bias in primate species with long interbirth intervals and non-seasonal breeding, such as hominid apes. However, the OSR of bonobos (*Pan paniscus*) is lower than that of chimpanzees (*Pan troglodytes*), which is thought to reduce competitive and aggressive male behaviors. The low OSR of bonobos is considered to result from the early resumption of female sexual receptivity during postpartum infertility and the receptivity that they continue to show until the late stage of pregnancy. In this study, we aimed to examine the early resumption of sexual receptivity by providing quantitative data on the resumption of maximal swelling (MS) in sexual skin and copulation, and changes in urinary estrone conjugate (E_1_C) concentrations during postpartum infertility in wild bonobos at Wamba in the Luo Scientific Reserve, Democratic Republic of the Congo. An analysis of 9 years of data revealed that females showed the first MS at 225.4 ± 132.7 days after parturition and performed the first copulation at 186.8 ± 137.5 days after parturition, both of which were in the early stage of postpartum infertility. The proportion of days with MS and the frequency of copulation steadily increased subsequently; however, the rate of increase gradually slowed approximately 42–48 months after parturition. There was a significant correlation between the proportion of days with MS and the frequency of copulation in each period for each female. We confirmed that E1C concentrations were significantly higher during the MS phase than during the non-MS phase. Data collected over 15 months on the E_1_C concentration during MS showed that it increased linearly from the early stage of lactation to the next conception. These results suggest that, although female bonobos do not usually conceive until 49.7 months after parturition, they resume MS and receptivity at a low level of E_1_C concentration during an early stage of postpartum infertility. This study of female bonobo receptivity and sex hormone changes during the postpartum non-fertile period provides important insights for examining the evolution of low OSR, which has been considered to contribute to peaceful social relationships among bonobos.

**Supplementary Information:**

The online version contains supplementary material available at 10.1007/s10329-021-00968-w.

## Introduction

In sexually reproducing animals, the operational sex ratio (OSR) is often used as a predictor for the intensity of mating competition. Meta-analytical studies have shown that the OSR has significant effects on competitive aggression, investment in courtship behavior, mate-guarding behavior, and copulation duration (Kvarnemo and Ahnesjo 1996; Weir et al. 2011). The OSR is affected by various factors, including the adult sex ratio, life history of both sexes, female reproductive rate, and environmental factors. For example, in primates with a non-seasonal breeding system that are more common in tropical areas (Heldstab et al. 2021), the male skew of the OSR increases as the receptive periods for the females are scattered across the year. This lowers the number of receptive females at any given time when compared to that with seasonal breeders, in which the receptive periods for females are concentrated in a limited mating season. Furthermore, in species with long interbirth intervals, like hominid apes (Emery Thompson and Sabbi 2019), the potential reproductive rate of females tends to be lower, which results in a largely male-skewed OSR (Mitani et al. 1996).

The chimpanzee (*Pan troglodytes*) is a good example of this, with a very long interbirth interval and absence of clear mating seasonality. The OSR in chimpanzees, which is calculated as the number of males per number of temporarily receptive females (Mitani et al. 1996), is estimated to be as high as 4.2 in the Mahale Mountains National Park, Tanzania and 12.3 in the Gombe Stream National Park, Tanzania [called “estrus sex ratio”, Furuichi and Hashimoto (2002)]. This might be one of the reasons for severe male aggression, which sometimes leads to fatal attacks (Fawcett and Muhumuza 2000; Watts 2004; Watts et al. 2006; Emery Thompson 2013; Kaburu et al. 2013; Wilson et al. 2014).

However, in the bonobo (*Pan paniscus*), the male skew of the OSR is lower than that in chimpanzees, because of the higher proportion of females showing receptivity [2.8 at Wamba (Furuichi and Hashimoto 2002)]. Consequently, inter-male aggression is more mitigated and mate choice by females is extended in bonobos (Kano 1992; Furuichi 1997, 2011; Wilson et al. 2014; Douglas et al. 2016; Gruber and Clay 2016; Sakamaki et al. 2018; Tokuyama et al. 2019). The higher number of receptive females also leads to other unique sociosexual features of the species, including high female social status and the development of close female associations (Kano 1992; White 1992; Parish 1996; Surbeck et al. 2010; Furuichi 2011; Ryu et al. 2015; Tokuyama and Furuichi 2016). Thus, the lower OSR greatly affects the social systems of bonobos.

Previous studies have suggested that the low male skew of the OSR in bonobos is due to the female tendency to show sexual receptivity during both postpartum infertility and pregnancy (Thompson-Handler et al. 1984; Kano 1992; Furuichi and Hashimoto 2002; Furuichi 2011). Regarding the receptivity during pregnancy, it has been reported that female bonobos continued to show maximal swelling (MS) and copulate after conception, for up to 6.5 months in the longest recorded case at Wamba (Furuichi and Hashimoto 2002) and for 3 months (range: 1–6 months, *N* = 6) at LuiKotale (Douglas et al. 2016). However, such post-conception swelling cycles have also been reported in chimpanzees (Tutin and McGinnis 1981; Takahata et al. 1996), and further studies are needed to determine whether such periods of receptivity are different between the two species.

The timing of the resumption of sexual receptivity during postpartum infertility, in contrast, does appear to show a large difference between the two species in the wild (Kano 1992; Furuichi and Hashimoto 2002; Deschner and Boesch 2007; Emery Thompson 2013). However, it is difficult to obtain sufficient quantitative data in the wild when the species of interest has relatively long interbirth intervals (IBIs). This is evidenced by the fact that there are only two studies for bonobos, and both report findings for small sample sizes [*N* = 6 for Wamba (Furuichi and Hashimoto 2002) and *N* = 5 for LuiKotale (Douglas et al. 2016)]. Furthermore, there are no quantitative data available on the timing of the resumption of MS and copulations or changes in the concentration of sexual hormones in females during their postpartum infertility. Therefore, in this study, we aimed to provide quantitative data on the resumption of sexual swelling and copulation after parturition to examine when female bonobos resume sexual receptivity using a 9-year dataset for nine female subjects at the Wamba study site in the Democratic Republic of the Congo. We also analyzed concentrations of urinary estrone conjugates (E_1_C) and pregnanediol glucuronide (PdG) in the nine female subjects over a 15-month period to examine the changes in the sexual hormones that occur with the resumption of receptivity.

This study reports the physical, behavioral, and hormonal changes that are related to the early resumption of sexual receptivity in female bonobos, which may lead to an increased number of receptive females and the mitigation of inter-male aggression. While the physiological mechanisms are different (Furuichi and Hashimoto 2002; Thornhill and Gangestad 2008; Furuichi 2019), female receptivity during postpartum infertility also increases the number of receptive females in humans, which is considered to have helped the formation of multiple pair-bonding mating units within a group (Lovejoy 2009; Chapais 2013; Furuichi 2020). Thus, this study of female receptivity during the infertile period in bonobos might provide important information for the study of the evolution of mating strategies and social organization in hominids.

## Materials and methods

### Study site and subjects

The bonobo field study was conducted at Wamba in the northern sector of the Luo Scientific Reserve, Democratic Republic of the Congo (DR Congo) (0°01′N, 22°34′E) (Kano 1992; Furuichi et al. 2012). Studies on bonobos began at Wamba in 1973. In 1976 the E group was chosen as the main study group due to their habituation, and each of the group members was identified that year. Between 1982 and 1983, the E group was split into E1, the main subject of this study, and E2 (Kuroda 1979; Kano 1982; Furuichi 1987; Furuichi et al. 2012). Field observations ceased from 1996 to 2002 because of the war in the DR Congo. Bonobos from the E1 group were provisioned until 1996, but there has been no provisioning since 2002 when research at Wamba was resumed. The size of the E1 group increased slightly during our main study period: from 26 individuals in 2007 (including nine adult males, seven adult females, three adolescent females, four juvenile and infant males, and three juvenile and infant females) to 38 individuals in 2016 (including seven adult males, nine adult females, four adolescent males, four adolescent females, six juvenile and infant males, and eight juvenile and infant females). The nine adult females were the subjects of this study, and they included six adult females who were present in 2007, one female (Nova) who was present in 2007 but only became a subject of this study in 2008 when she first gave birth, and two other females (Fuku and Otomi) who immigrated into the study group in 2008 and became subjects of the study in 2010 when they first gave birth (Table 1). Further details of the study site and group have been described elsewhere (Kano 1992; Furuichi et al. 2012).

**Table 1:** Female bonobo data profiles used in the analyses of this investigation

			Sexual swelling, copulation, and parturition data for the 2007–2016 study period								Hormonal analysis data for the 2013–2015 study period					
Subject females	Estimated birth year^1^	Primiparous/multiparous	# Observation days	# Days in which each female was observed (%)	# Parturition	# Analyzed 6-month periods after parturition^3^	# Days in the analyzed 6-month periods	# Days with maximal swelling in the analyzed 6-month periods	# Observed copulations on all observation days	# Copulations in the analyzed 6-month periods	# All analyzed urine samples	# Cycles with estimated ovulation	# 1-month periods after parturition^4^	# Urine samples in all 1-month periods after parturition^4^	# Analyzed 1-month periods^5^	# Urine samples during maximal swelling in the analyzed 1-month periods^5^
Nao	1971	m	2521	2039 (80.9)	1	15	1693	456	216	204	120	3	5	46	4	28
Kiku	1974	m	2521	2176 (86.3)	2	14	1779	550	126	115	44	-^6^	3	43	1	9
Hoshi	1983	m	2521	2098 (83.2)	2	11	1402	381	239	183	36	-^6^	3	32	2	9
Yuki	1983	m	2521	1993 (79.1)	2	15	1668	751	126	109	31	-^6^	2	29	2	11
Jacky	1988	m	2521	1990 (78.9)	1	14	1637	762	441	358	60	4	11	60	5	33
Sala	1992	m	2521	2113 (83.8)	1	8	1033	317	500	314	114	2	12	111	8	42
Nova	1995	p → m^2^	2248	1719 (76.5)	2	13	1461	467	310	269	54	-^6^	2	23	1	5
Fuku	1997	p → m^2^	1829	1300 (71.1)	2	10	1113	267	362	332	139	4	9	133	8	77
Otomi	1998	p → m^2^	1858	1260 (67.8)	1	7	830	179	431	290	62	1	-^7^	-^7^	-^7^	-^7^
Total					14	107	12616	4130	2751	2174	660	14	47	477	31	214
Average			2340.1	1854.2 (78.6)	1.6	11.9	1401.8	458.9	305.7	241.6	73.3	2.8	5.9	59.6	3.9	26.8
SD			295.5	350.1 (6.0)	0.5	3.0	336.0	202.2	137.9	92.5	40.1	1.3	4.2	40.6	2.9	24.3

^1^Birth years were estimated from the body size and the size and shape of the sexual skin when these females were first observed in the study group

^2^The subject females were primiparous but became multiparous during this study. There were fewer observation days for Nova, Fuku, and Otomi than the other subjects, as they first gave birth in the middle of the study period

^3^Excluding 6-month periods of nulliparity, periods during pregnancy, and periods for Sala and Otomi after stillbirth and before next conception. Periods with ≤ 80 days of observations were also excluded

^4^Excluding 1-month periods during pregnancy, and periods for Otomi after stillbirth and before next conception

^5^Number of above-mentioned 1-month periods that had three or more urine samples during maximal swelling

^6^Females who were pregnant or at very early stage of nursing throughout the study period for hormonal analysis

^7^No 6-month period was included in the analysis of E_1_C concentration because Otomi was pregnant or after stillbirth throughout the study period

### Observations and sample collection

The results of the current study originated from three datasets as follows: (1) we used a 34-year dataset between 1976 and 2016 for the estimation of IBIs; (2) we used a 9-year dataset between 2007 and 2016 for the analyses of occurrences of MS and copulation after parturition; (3) we used a 15-month dataset between 2013 and 2015 for the analyses of changes in sexual hormones after parturition. The female subjects and the data analyzed for the second and third datasets are listed in Table 1.

### Interbirth intervals

Researchers and research assistants have recorded all bonobo births in the E group and E1 group (after the split) from the beginning of field observations in 1976, except during the absence of researchers from 1996 to 2002 owing to war in the DR Congo. We calculated the IBI using data from 1977 to 1996 (*N* = 13) and from 2002 to 2016 (*N* = 10); we used the intervals between the previous birth and the subsequent birth that was confirmed while the offspring of the previous birth was still alive. We observed one case of stillbirth for the female named Otomi in 2014, and another possible case for a female named Sala in 2009, though it was not confirmed whether it was a stillbirth or the death of a newborn baby. We did not include the IBIs before these two cases in the analysis.

### States of sexual swelling and the proportion of maximal swelling

On the days of observation from September 2007 to June 2016, researchers and research assistants followed one ranging party of the group that was first found on each day, the members of which sometimes changed flexibly, and recorded the states of sexual swelling of females found in the party. The daily state of the anogenital region wrinkling was scored according to an established method used since 1985 (Furuichi 1987; Ryu et al. 2015). It was assigned to one of the following three categories: (1) non-swelling (sw1), in which the sexual skin is highly wrinkled and sways when the animal is walking; (2) intermediate swelling (sw2), in which the sexual skin is turgid, but small wrinkles are visible on its surface; and (3) MS (sw3), in which the sexual skin appears firm and lustrous without wrinkles. All the female subjects experienced all three swelling states. To avoid observation bias, researchers and research assistants recorded the observations independently, and scores were determined by consensus after discussions at the daily evening meetings. By doing so, researchers and research assistants re-assessed and adjusted their standards for the daily scoring. A similar categorical evaluation of the state of the sexual skin by visual observation has been conducted in other bonobo studies (Surbeck et al. 2012; Douglas et al. 2016).

The female subjects were observed, in the parties that we followed, for 78.6 ± 6.0% of all observation days (*N* = 9 females, Table 1). The main reason that females were sometimes not observed was that the study group split into two or more mixed-sex parties (see an example of the year 2015, Online Resource 1). Occasionally, a few females were not observed on days when all the males were observed. This seemed to be because the females stayed in the periphery of the mixed-sex parties, as the rates of observation were lower for the three young low-ranking females (Table 1). Although we cannot exclude the possibility that they may have ranged independently as single females or stayed in all-female parties, we did not observe such parties throughout the study period. Overall, within the days when all males were observed in 2015 (*N* = 146), the sum of the days on which each female was not observed was 57, which was only 4.3% of the total number of female-days (*N* = 9 females × 146 days = 1314). Therefore, even if there was a tendency for females in the non-MS phase (sw1 and sw2) to more likely range apart from the males, their absence did not provide a substantial bias to the data.

Although each female was observed quite regularly, it was still difficult to determine the onset and the end of the MS phase due to the gaps in observations. Therefore, to examine the changes in sexual swelling, instead of the length or frequency of the MS phase, we examined the changes in the proportion of days on which each female showed MS when compared to the total observation days for the female. We calculated the proportion of MS in each 6-month period since the day of parturition (0–6 to 66–72 months). We employed the 6-month time frame for two reasons: (1) Females are in MS phase at intervals of ca. 40 days; therefore, the proportion of MS will be largely biased by the number of MS phases included in each time frame if we employed shorter time frames. (2) Large seasonal fluctuations of rainfall and fruit production (Furuichi et al. 2015) may affect female sexuality; therefore, we wanted to employ long time frames to reduce the influence of such environmental factors. We estimated the date of conception from the date of next parturition and the mean gestation period of bonobos (see Results section), and the duration of pregnancy was excluded from this calculation. We excluded the data for females who experienced stillbirth or death of a newborn baby, from the earliest possible date of conception of the dead infant to the date of next conception, because we were unsure about the effects of stillbirth on the physiological state of females. To avoid biases due to a small sample size, we excluded the 6-month periods in which a female subject was not observed for more than 80 days, which is regarded as double the mean menstrual cycle (Furuichi 1987; Douglas et al. 2016). A total 107 6-month periods were analyzed [11.9 ± 3.0 (SD) per female, Table 1].

### Frequency of copulation

Researchers and two research assistants recorded observed copulations [*N* = 2751, 305.7 ± 137.9 (SD) per female, Table 1] using the sampling of all occurrences method (Altmann 1974). The research assistants moved around within the ranges of the observed parties to check for the presence of the members in each 1 h observation window (Hashimoto et al. 2001). Furthermore, copulations were usually performed in the trees and rather easily noticed from a distance. Although the copulations that the assistants observed may have been biased to those who were in the central part of the party, this is an unavoidable limitation of the field method employed in the current study. The data on the copulations that were observed only by the researchers were not included because the number of researchers varied from day to day, and some of them focused on a few specific individuals for their own research purposes. We calculated the frequency of copulation of each female: the number of observed copulations per day on which each female was observed in each of the analyzed 6-month periods since the day of parturition. We employed the 6-month time frame for the same reasons as those for the proportion of MS. We analyzed 2174 copulations for the subject females [241.6 ± 92.5 (SD) per female, Table 1] that were observed during the analyzed 6-month periods (*N* = 107).

Although the frequency of copulation might be affected by the number of males present in the party, the males in the study group were observed in the parties that were followed quite regularly (Online Resource 1). There was only a small variation in the mean number of males that were found with each female during the daily observations [5.9 ± 0.6 (SD)] during the analyzed 6-month periods. Furthermore, there was no significant correlation between the mean number of males and the frequency of copulations (Pearson’s correlation test, *N* = 107, *t* = −1.53, *df* = 105, n.s.). Therefore, we assumed that the temporal presence or absence of male members in the observed parties did not affect the frequency of copulation in each 6-month period.

### Hormone analysis

We collected urine samples from September 2013 to February 2014, July 2014 to September 2014, and November 2014 to April 2015 for the analyses of the timing of ovulations and changes in concentration of sexual hormones after the parturition. The urine samples were collected after the bonobos had left the area, to maintain a distance of at least 5 m between the collector and the bonobos. We dipped filter papers (Whatman #1 Ø 5.5 cm, GE Healthcare Life Sciences, UK) in the urine that had dropped on the leaves of terrestrial plants or small trees and placed them in separate plastic zipper bags. Urine contaminated with feces was not collected. At the base camp, we stored the filter papers in plastic bags with their zippers opened in a dry box containing 500 g silica gel for 1 week. After 1 week, the completely dried filter papers were stored in sealed plastic bags in a dark place at room temperature (20–28 °C) for less than 6 months. The dried samples were transported to the Kyoto University Primate Research Institute, Japan, where they were kept in a freezer at –20 °C until the hormone analysis.

In total, we used 660 urine samples from the nine female subjects to measure urinary steroid metabolites [73.3 ± 40.1 (SD) per female, Table 1]. We extracted urine from the filter paper using a method developed by Mouri and Shimizu (2021). In brief, we placed each piece of filter paper in an extraction tube with 2 mL of deionized water, which was shaken (160 rpm for 180 min) to elute the urinary content into the solution. Mouri and Shimizu (2021) confirmed that the profiles of E_1_C and pregnanediol glucuronide (PdG) that were derived from urine that was extracted from filter papers that had been preserved for 1 year was almost the same as that derived from the frozen samples of the original urine, and both provided the same expected dates of ovulation. Mouri and Shimizu (2021) also confirmed that the concentrations of E_1_C and creatinine on the filter paper were stable for 1 year at room temperature.

We analyzed the concentrations of E_1_C and PdG using a protocol that was developed for a study on Japanese macaques (Shimizu and Mouri 2018). Mouri and Shimizu (2021) used this protocol for the analysis of chimpanzee urine and confirmed its validity. In the current analyses with bonobo urine, the slope of the displacement curves produced by serial dilutions of eluents from the filter papers was parallel to the slope obtained from the corresponding EIA standard curves (*P* values ranged from 0.155 to 0.337 for E_1_C and 0.255 to 0.730 for PdG). The mean ± SD recovery for the known amounts of steroid hormone added to the eluents from the filter papers for E_1_C and PdG were 106.2 ± 13.6% and 92.1 ± 25.1%, respectively. The inter-plate coefficient of variation (CV) for E_1_C was 10.79% when using the control with 1.00 ng/mL concentration of E_1_C (assay range: 0.01–10 ng/mL). The inter-plate CV for PdG was 15.83% when using the control with a 200 ng/mL concentration for PdG (assay range: 1–1000 ng/mL). Intra-assay CVs were 5.66% for E_1_C and 6.66% for PdG. A total of 660 samples from the nine females were used for the examination of the inter-plate CV and intra-assay CV (Table 1). Concentrations of E_1_C and PdG were compensated for with creatinine concentrations measured using the Jaffe reaction (Taussky and Kurzmann 1954).

Using methods described in previous studies (Douglas et al. 2016), the occurrence of the ovulation date was estimated based on a sustained urinary PdG increase over two standard deviations above the baseline, which was defined as the mean urinary PdG concentrations of the preceding 10 days. We did not estimate the ovulation date for the cycles in which fewer than three samples were collected within 10 days before the sustained urinary PdG increase.

To assess the levels of the E_1_C concentrations that caused sexual swelling, we compared the value of the E_1_C concentrations with the state of sexual swelling of the female on the day of the sample collection [*N* = 477, 59.6 ± 40.6 (SD) per female, Table 1]. To assess the changes in E_1_C concentrations after parturition, we analyzed the median E_1_C concentration during MS in each 1-month period [*N* = 47, 5.9 ± 4.2 (SD) per female, Table 1]. We did not use the 6-month period for this analysis because there were gaps within the 15-month sample collection; some 6-month periods included only a few months of the sample collection. We omitted the 1-month periods of some females with less than three samples during the MS.

### Data analysis

We used R 4.0.2 (R Core Team 2020) with RStudio 1.3.1056 (2009–2020 RStudio, PBC) for all data analysis. We used the lmer and glmer functions of the lme4 package (Bates et al. 2014) and lmerTest (Kuznetsova et al. 2017) to fit our models to the data and to examine the *p* values.

For the generalized linear mixed model (GLMM) analysis, to assess the effect of the number of months after parturition on the proportion of days in MS over the 9-year study period (model 1, Table 2), we used a glmer function with a binomial error structure linked by a logit function. The response variable was the binomial value of the number of days in MS and the number of days in non-MS linked with the cbind function. For the analysis of the effect of months after parturition on the frequency of copulations in the 9-year study period (model 2, Table 2), we used a glmer function with a Poisson error structure linked to the log function. The response variable was the number of copulations observed in each 6-month period with an offset of the number of observation days in the same period. The ID of each female was included as a random variable in models 1 and 2. The correlation between the proportion of MS days and the frequency of copulations (number of copulations divided by number of observation days) in each 6-month period was analyzed using Pearson’s correlation test.

**Table 2:** Parameter estimates for generalized linear mixed models

(1) Effect of the number of months since parturition on the proportion of MS days					
Unit: each 6-month period from parturition					
Family: binomial (logit)					
Formula: proportion of MS days and non-MS days ~ months from parturition + months from parturition squared + (1|female ID)					
	Estimate	Std. Error	*z* value	Pr( >|*z*|)	
(Intercept)	−0.75623	0.0928	−8.377	< 2e–16 ***	
Months from parturition	−0.81452	0.03102	26.254	< 2e–16 ***	
Months from parturition squared	−0.27692	0.02664	−10.395	< 2e–16 ***	
(2) Effect of the number of months since parturition on the frequency of copulation					
Unit: each 6-month period from parturition					
Family: Poisson (log)					
Formula: number of copulations ~ months from parturition + months from parturition squared + offset [log (number of observation days)] + (1|female ID)					
	Estimate	Std. Error	*z* value	Pr( >|*z*|)	
(Intercept)	−1.90034	0.23081	−8.233	< 2e–16 ***	
Months from parturition	0.69700	0.03161	22.05	< 2e–16 ***	
Months from parturition squared	−0.21739	0.02994	−7.261	3.83e–13***	
(3) E1C concentrations at different swelling states					
Unit: each urine sample					
Formula: log (E_1_C concentration + 0.5) ~ swelling phase + (1|female ID)					
	Estimate	Std. Error	*df*	*t* value	Pr( >|*t*|)
(Intercept)	2.17127	0.11445	8.28045	18.97	4.08e−08***
sw3 vs sw2	−0.38446	0.07323	473.524	−5.25	2.30e−07***
sw3 vs sw1	−1.33268	0.09018	469.982	−14.78	< 2e−16 ***
(4) Effect of the number of months since parturition on E1C concentrations during MS					
Unit: each 1-month period from parturition					
Formula: log (median E_1_C concentration) ~ months from parturition + months from parturition squared + (1|female ID)					
	Estimate	Std. Error	*df*	*t* value	Pr( >|*t*|)
(Intercept)	2.22171	0.09914	28	22.41	< 2e−16 ***
Months from parturition	0.29557	0.10081	28	2.932	0.00664 **
Months from parturition squared	−0.0757	0.10081	28	−0.751	0.45892

The relationship between E_1_C concentration and swelling status was examined using a lmer function (model 3, Table 2). The response variable was the E_1_C concentration of each urine sample in all 1-month periods in the 15-month study period except during pregnancy and after stillbirth, and the predictor was the three states of sexual swelling of the female on the day of the sample collection (N =  477). The response value was transformed using an equation of log (E_1_C + 0.5) and confirmed for normality using the Shapiro–Wilk test (W = 0.99, n.s.). A lmer function was used for the analysis of the effect of months after parturition on the E_1_C concentration during MS in the 15-month study period (model 4, Table 2). The response variable was the median value of the E_1_C concentrations for each female during MS in each analyzed 1-month period that had three or more urine samples. The median value of E_1_C was largely skewed toward zero and was therefore log-transformed and it was confirmed for normality using the Shapiro–Wilk test (*W* = 0.94, n.s.). The ID of each female was included as a random variable in models 3 and 4.

For models 1 and 2, we included the duration from parturition to the midpoint of each 6-month period as a predictor. Since the effects of the duration after parturition on the response variables of the above three models seemed to be non-linear, we also tested the models by including duration squared (quadratic term) as a predictor to improve reliability. For this purpose, we z-transformed the duration after parturition to a mean of zero and a standard deviation of one. In all three models, we first checked the significance of the differences between the full model including all predictors and the null model including only a constant as a predictor using analysis of variance (ANOVA) in R (Dobson and Barnett 2018). When these differences were significant, we compared the model with linear and quadratic terms to the model with only a linear term using ANOVA to select the models with smaller Akaike information criterion (AIC) values, and we checked the significance of the contributions of each predictor in the selected models. We checked variance inflation factors (VIF) (Queen et al. 2002) using the function of vif from the package car (Fox and Weisberg 2018). The maximum VIF was 1.57 for model 1, 1.34 for model 2, and 1.26 for model 3; thus, the multicollinearity between the predictor variables was not of great concern in the analysis (Zuur et al. 2010).

## Ethical statement

All methods used to collect observational data and urine samples were noninvasive. These methods complied with the Guidelines for the Studies of Wild or Wild-origin Primates of the Primate Research Institute, Kyoto University. Study methods and the export of urine samples were approved by the Ministry of Scientific Research and Technology of the DR Congo.

## Results

### Interbirth interval, gestation, and postpartum infertility

To estimate the duration of postpartum infertility from parturition to the next conception, we calculated the IBI for the bonobos in the E1 group. The mean IBI between 1977 and 1996 and between 2002 and 2016 was 57.7 ± 9.6 (SD) months (range: 43–72, *N* = 23 IBIs from 13 females). There was no significant difference in the IBI before 1996 [58.5 ± 11.6 (SD) months, range: 43–72, *N* = 13 IBIs from seven females] and after 2002 [56.5 ± 6.6 (SD) months, range: 50–71, *N* = 10 IBIs from eight females; Wilcoxon exact test, *W* = 73.0, *p* = 0.64], suggesting that the IBI was not influenced by the provisioning that was conducted until 1996. Two gestation periods of 230 and 242 days were estimated via hormonal analysis (see Online Resource 2 for the methods and more detailed information). These were similar to the 229-, 234-, and 238-day gestation periods reported from captive bonobos (Heistermann et al. 1996). If we consider that the gestation period is the mean value of the two periods of this study (236 days, 7.9 months), bonobos at our study site are expected to be in postpartum infertility for 49.7 months on average.

### Resumption of MS and the changes in its proportion after parturition

The mean duration from parturition to the first day of MS was 225.4 ± 132.7 (SD) days (range: 32–516, *N* = 14 cases from nine females, Table 1). However, this value might be underestimated as several females showed MS on a single isolated day at an early stage of lactation. If these single days were excluded, the mean duration from parturition to the earliest MS was 244.0 ± 127.9 (SD) days (range: 108–550, *N* = 14 cases from nine females).

The proportion of MS days to total observation days for each female was 33.5 ± 8.6 (SD) % (*N* = 9 females). This proportion of MS days was similar to the proportion of those calculated from the cycle length and length of the MS phase that were reported from previous studies [34.8% (Furuichi 1987), 26.0% (Douglas et al. 2016)]. This comparison supports our assumption that the presence of females in the observed parties was not largely biased by the status of their sexual swelling.

**Fig. 1:**
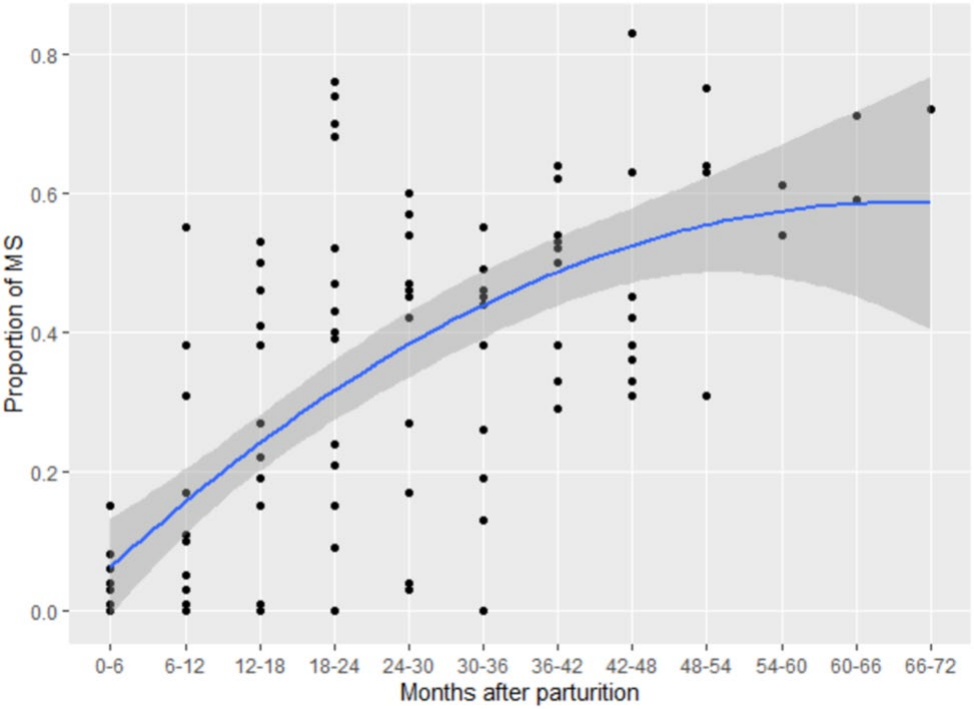
Changes in the proportion of observation days with maximal swelling (MS) with increase in time after parturition (model 1). Each dot represents one female in each 6-month period (*N* = 113 for 21 interbirth intervals of nine females). The *x*-axis shows the duration in months from parturition to the midpoint of each 6-month period. The *y*-axis shows the proportion of MS in each 6-month period. Some females have two dots in the same period because they had two parturition events during the study period. The blue line shows the fit of the model including the linear and squared terms of the number of months after parturition, and the grey shading shows the 95% confidence area of the fitted line

ANOVA of the proportion of MS showed that a full model including both linear and squared terms for the duration after parturition was significantly different from the null model (*χ*^2^ = 830.24, *df* = 2, *p* < 0.0001). The model including both linear and squared terms was selected (ANOVA, AIC = 1963.0) as it was better than the model including only a linear term (AIC = 2069.4), and GLMM analysis showed that both terms had significant effects on the proportion of MS (model 1, Table 2). These results indicate that the proportion of MS increased with time, but the rate of increase changed with time (Fig. 1). It steadily increased from the very early stage of lactation and continued to increase until the next conception, although the rate of increase gradually decreased from approximately 42–48 months.

### Resumption of sexual receptivity and changes in frequency of copulation

The duration from parturition to the first observed copulation was 186.8 ± 137.5 (SD) days (range: 30–435 days, *N* = 14 cases from nine females). Twelve out of the 14 observed copulations occurred in the non-MS phases. The duration was 344.4 ± 135.5 (SD) days (range: 30–435 days, *N* = 14) if copulations in the non-MS phase were excluded. Copulations were most likely observed at MS (5.9% at sw1, 25.0% at sw2, and 70.1% at sw3, *N* = 2751).

**Fig. 2:**
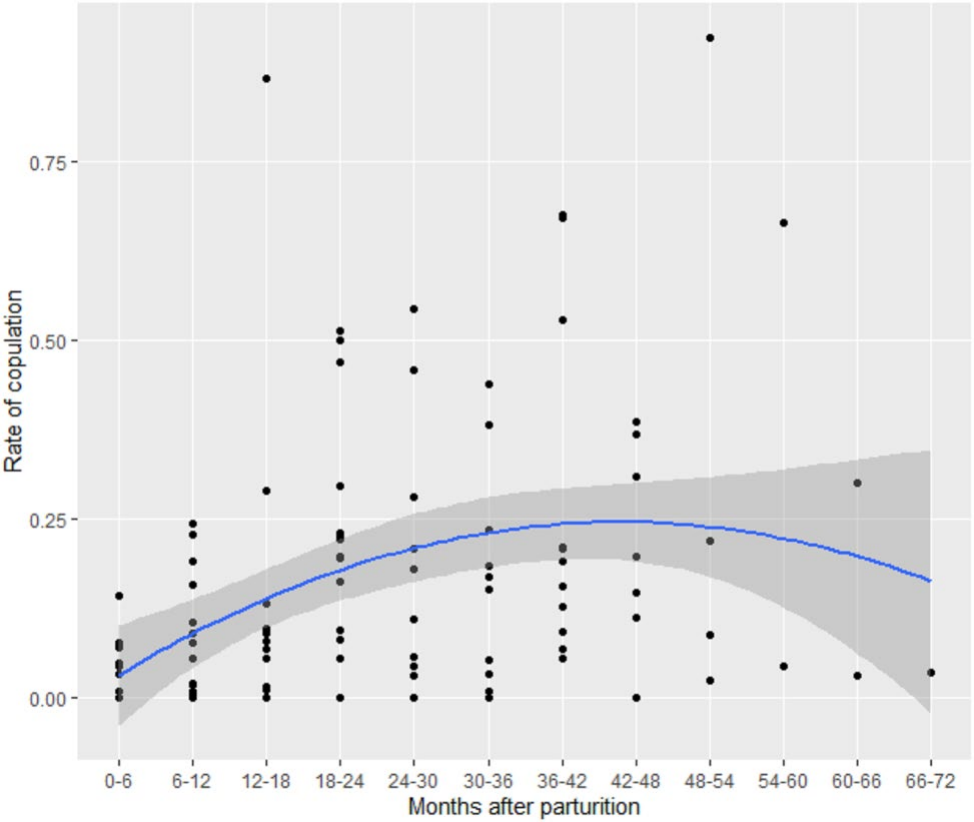
Changes in the frequency of copulation with increase in time after parturition (model 2). Each dot represents one female in each 6-month period (*N* = 113 for 21 interbirth intervals of nine females). The *x*-axis shows the duration in months from parturition to the midpoint of each 6-month period. The *y*-axis shows the number of copulations observed per day of observation in each 6-month period. Some females have two dots in the same period because they had two parturition events during the study period. The blue line shows the fit of the model including the linear and squared terms of the number of months after parturition, and the grey shading shows the 95% confidence area of the fitted line

ANOVA of the frequency of copulation showed that a full model including both linear and squared terms for the duration after parturition was significantly different from the null model (*χ*^2^ = 601.07, *df* = 2, *p* < 0.0001). The model including both linear and squared terms was selected (ANOVA, AIC = 1561.0) as it was better than the model including only a linear term (AIC = 1615.2), and GLMM analysis showed that both terms had significant effects on the frequency of copulation (model 2, Table 2). These results indicate that the proportion of copulation and the rate of increase changed with time (Fig. 2). The frequency of copulation steadily increased from an early stage of lactation and reached the highest level after approximately 42–48 months. This pattern was similar to the pattern of increase in the proportion of MS though the latter did not show a clear peak, and there was a significant correlation between the proportion of MS and the frequency of copulation in each 6-month period (Pearson’s correlation test, *N* = 107, cor = 0.44, *t* = 4.97, *df* = 105, *p* < 0.0001).

### Hormone profile and changes in E_1_C concentrations after parturition

**Fig. 3:**
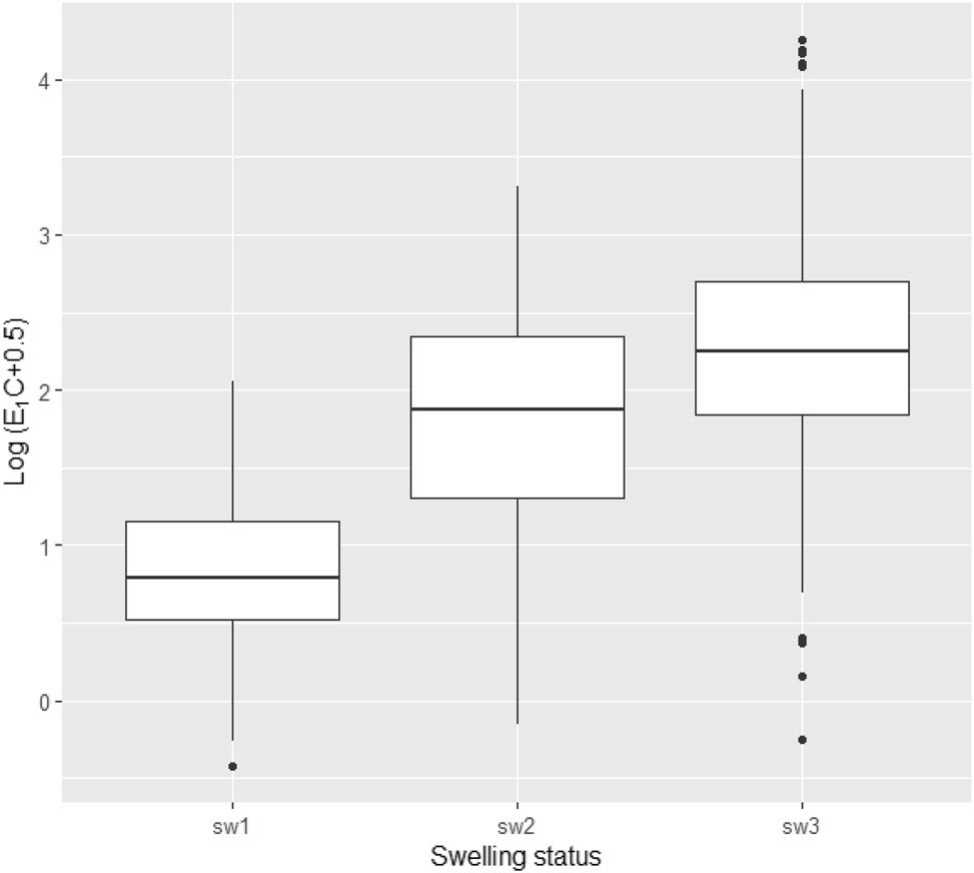
E_1_C concentrations in each swelling status (model 3). The unit of analysis is each urine sample of each female. The middle lines represent the median values of all urine samples in each swelling status. Boxes represent quartile ranges; whiskers represent maximum and minimum values excluding outliers shown by dots

As we could not estimate the date of ovulation in some cycles due to sampling gaps, we could only estimate the timing of ovulation in 14 cycles for five of the females (Table 1). These cycles consisted of 11 non-conception and three conception cycles. The changes in E_1_C and PdG in the 11 non-conception cycles followed a general pattern previously reported for bonobos (Heistermann et al. 1996; Douglas et al. 2016) (Online Resource 3). This suggests that our methods for collection of urine samples and analyses of sexual hormones were effective for monitoring the changes in concentration of these hormones in wild bonobos. The 14 ovulation cycles occurred at 42.4 ± 11.5 (SD) months after parturition (range: 24–63 months). Ovulation was not detected in four females because they were pregnant or at a very early stage of nursing (less than 21 months after parturition). Thirteen of the 14 ovulations (92.9%) occurred toward the end of the MS (Online Resource 4). The E_1_C concentration was higher in MS than in non-MS (sw3 > sw2, sw3 > sw1, *N* = 477, model 3, Table 2, Fig. 3), suggesting that it is one of the factors that may cause sexual swelling.

**Fig. 4:**
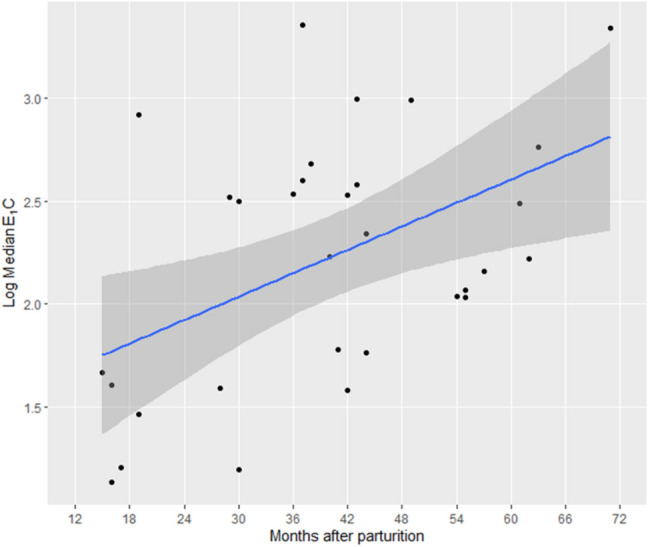
Changes in E_1_C concentrations with increase in time after parturition (model 4). The *x*-axis shows the duration in months from parturition. The *y*-axis shows the median E_1_C value during MS for a given female in a given 1-month period (*N* = 47 for eight interbirth intervals of eight females). We log-transformed the value to fit the normal distribution. The blue line shows the fit of the model including the linear term of the number of months after parturition, and the grey shading shows the 95% confidence area of the fitted line

ANOVA of the E_1_C concentrations during MS showed that a full model including both linear and squared terms of the months after parturition was significantly different from the null model (*χ*^2^ = 7.5, *df* = 2, *p* < 0.05). The model including only a linear term was selected (ANOVA, AIC = 56.6) as it was better than the model including both linear and squared terms (AIC = 58.0), though the difference in AIC was less than the significant level of 2 (Burnham and Anderson 2004). GLMM analysis showed that only the linear term had a significant effect on the E_1_C concentration during MS (model 4, Table 2). As shown in Fig. 4, the E_1_C concentration during MS steadily increased from an early stage of lactation to the next conception.

## Discussion

### Changes in the proportion of MS, frequency of copulation, and E_1_C concentrations during MS after parturition

Although female bonobos are known to show sexual receptivity during postpartum infertility (Thompson-Handler et al. 1984; Kano 1992; Furuichi and Hashimoto 2002), there were very limited data available concerning the timing of the resumption and changes in the MS and copulation during this period. As the resumption of MS and copulation are infrequent events for each female, this study examined these issues using a long-term dataset (9 years) on wild bonobos at Wamba. Females started showing MS at 225.4 ± 132.7 days (ca. 7.5 months, *N* = 14) or at 244.0 ± 127.9 days if MS’s of single days are excluded (ca. 8.1 months, *N* = 14), which was similar to previous reports from Wamba [within 1 year (Kano 1992); < 8 to 15 months, *N* = 6 (Furuichi and Hashimoto 2002)] and LuiKotale [7.0 ± 4.8 months, *N* = 5 (Douglas et al. 2016)]. The first copulation was observed at 186.8 ± 137.5 days after parturition (ca. 6 months). Although female bonobos perform copulations mostly during the MS phase, they sometimes perform copulation during the non-MS phase and those might be social rather than sexual behaviors (Thompson-Handler et al. 1984; Furuichi 1987; Dahl et al. 1991; Ryu et al. 2015). Therefore, to examine the resumption of apparent sexual receptivity, we examined the timing of the first copulation by excluding copulations during the non-MS phase; this was observed at 344.4 ± 135.5 days after parturition (ca. 11 months). Although the first copulations occurred a little earlier than the first MS and the first copulations during the MS phase occurred a little later than that, it was apparent that female bonobos resumed sexual receptivity during the early stage of postpartum infertility.

Both the proportion of MS and frequency of copulation steadily increased with time from a very early stage after parturition, but the rate of increase gradually slowed 42–48 months after parturition. Because the interbirth interval was 57.6 months and the gestation period was 7.9 months, bonobos in this study conceived 49.7 months on average after parturition. This means that females started to show MS 42.2 months before and performed first copulation 43.7 months before (or 38.7 months before if copulations during non-MS were excluded), and the proportion of MS and the frequency of copulation steadily increased until, the average timing of next conception. The existence of a significant correlation between the proportion of MS and the frequency of copulation in each 6-month period suggested that female bonobos recovered both attractivity and receptivity together from the early stage of postpartum infertility.

Such tendencies differ from those of chimpanzees. Although western chimpanzees tend to resume sexual swelling earlier (24, 21.6 ± 14.4, and 28.8 ± 18.0 months after parturition in different reports from Taï) than eastern chimpanzees (46.8 months in Gombe, 55.2 months in Mahale, 52.8 months in Kanyawara) (Deschner and Boesch 2007), bonobos resume sexual swelling more than 14 months earlier than western chimpanzees and more than 44 months earlier than eastern chimpanzees. Although the interbirth interval in chimpanzees is slightly longer than that in bonobos, there is no significant difference between the species (Furuichi and Hashimoto 2002; Emery Thompson and Sabbi 2019). Moreover, the weaning period does not differ between the two species, as it occurs at approximately 3.5–5 years in bonobos (Kano 1992; Hashimoto 1997; Lathouwers and Elsacker 2006) and 4–5 years in chimpanzees (Clark 1977; Lathouwers and Elsacker 2006; Emery Thompson et al. 2012; Smith et al. 2013). The difference between the species, therefore, is that chimpanzees do not show MS or sexual receptivity during postpartum infertility and become pregnant shortly after they resume MS (Emery Thompson 2013), while bonobos show MS and sexual receptivity during the early stage of postpartum infertility but become pregnant much later than these occurrences.

To examine the hormonal occurrences during the resumption of MS and sexual receptivity, we examined the changes in the concentrations of the sexual hormones in female subjects during a 15-month period in the above-mentioned long-term study period. While the earliest ovulation that occurred for a female chimpanzee was 25 months after parturition (Deschner et al. 2004), it was confirmed to be 24 months in this study for the bonobos. The E_1_C concentration during MS steadily increased from the early stage postpartum infertility to the next conception. Douglas et al. (2016) showed that the relationship between sexual swelling and E_1_C levels was not necessarily close; however, the fact that E_1_C concentrations showed a similar pattern of increase as the proportion of MS and frequency of copulation during postpartum infertility may suggest that the increase in E_1_C concentrations influenced the resumption of MS and receptivity. In chimpanzees, the mean E_1_C levels during the peri-ovulatory period increased significantly as the number of cycles to conception decreased (Deschner et al. 2004), which means that the E_1_C level also gradually increased until the timing of conception in chimpanzees.

These comparisons suggest that for both bonobos and chimpanzees, several important physiological changes occur at approximately the time of weaning when mothers become prepared for the next conception. Therefore, an important difference between the two species is that female chimpanzees resume sexual swelling cycles when they become almost ready for conception with a high level of E_1_C concentration, whereas female bonobos resume this state very early during the non-fertile period when the E_1_C concentration is still low. While further studies on the background physiological mechanisms are required, there could be certain differences in the sex skin reactions to estrogen rises between the two species. For example, Douglas et al. (2016) suggested that, in bonobos that displayed sexual swellings that were decoupled from the ovulatory cycles, estrogen and progesterone receptor sensitivity and density of sexual swellings may be different, or may fluctuate in a different way in relation to hormone excretion, in contrast to other species. Such a mechanism may explain not only the sexual swelling decoupled from the ovulatory cycles but also the early resumption of sexual swelling at a low level of E_1_C concentration in bonobos.

Although the 9-year dataset that we used for the analyses of changes in the proportion of MS and frequency of copulations covered only two IBIs, it is the longest available data that included 14 births by all nine females in the group. Therefore, it makes an important contribution to our knowledge concerning the early resumption of sexual receptivity by female bonobos. However, the 15-month dataset used for the analyses of changes in E_1_C concentrations and relationships between E_1_C concentrations and sexual swelling was too short to draw conclusions on the relationships between the resumption of sexual receptivity and the changes in E_1_C concentrations. We present this result in this paper to provide insights into the relationship because there has been no other study concerning this issue in wild bonobos. However, we need more long-term continuous monitoring data on the changes in E_1_C concentrations from parturition to the next conception in specific females.

### Influence of sexual receptivity during postpartum infertility on the mating system

The early resumption of female sexual receptivity lowers the proportion of adult males to receptive adult females (OSR) in bonobos, by increasing the number of females who show receptivity at the same time. A study of the bonobos at Wamba reported that as many as 3.1–4.1 females showed receptivity in a group, including 7–8 females, and the proportion of adult males to receptive adult females was 1.5–2.3. These values were much lower than those calculated for eastern chimpanzees in Mahale (4.2) and Gombe (12.3) (Furuichi and Hashimoto 2002). Such a lower proportion of males to receptive females in bonobos might play an important role in mitigating aggressive male-male competition over mating (Furuichi 2011). Agonistic interactions among males over access to receptive females are seldom observed in bonobos at Wamba (Furuichi 1997; but also see Surbeck et al. 2010). Surbeck et al. (2012) showed that the testosterone levels of high-ranking males were lower than those of low-ranking males and that they did not increase in the presence of potentially fertile females at LuiKotale. The authors suggested that amicable relationships between the sexes, rather than aggressive interactions, mediate the physiological reactivity of males during periods of male competition. Such relationships between female receptivity during infertile periods and male–female mating strategies might also be observed in chimpanzees. In western chimpanzees in Taï, where females resume sexual swelling earlier and have a greater number of cycles to conception than eastern chimpanzees (Deschner and Boesch 2007), male sexual coercion was relatively infrequent and did not seem to constrain female mate preferences (Stumpf and Boesch 2010).

The proportion of males to receptive females is also very low in humans, which seems to help in the formation of multiple pair-bonding units as subunits of a social group (Furuichi 2006; Thornhill and Gangestad 2008; Lovejoy 2009; Chapais 2013). While bonobo females extend the receptive period with sexual swelling into the infertile period, human females evolved continual receptivity in the infertile periods during lactation, pregnancy, and the non-periovulatory period within a menstrual cycle (Thornhill and Gangestad 2008). Despite these physiological differences, both species share a common feature of the loose relationship between fertility and receptivity of females. Although there are several papers that discuss how the extended period of sexual receptivity caused by the early resumption of receptivity affects the sociosexual relationships in bonobos (Kano 1992; Wrangham 1993; Furuichi 2011), we need to conduct further studies on the hormonal and genetic backgrounds related to this feature to understand the process and the ultimate cause of divergence between bonobos and chimpanzees. Such studies may also provide important hints on the absence of sexual swelling and on the evolution of continual receptivity in human females and contribute to our understanding of the role of sexuality in the evolution of social systems in hominids.

## Supplementary Information

Below is the link to the electronic supplementary material.

Supplementary file1 (DOCX 655 KB)
