# Correction to: Physical, behavioral, and hormonal changes in the resumption of sexual receptivity during postpartum infertility in female bonobos at Wamba

**DOI:** 10.1007/s10329-022-01007-y

**Published:** 2022-08-09

**Authors:** Chie Hashimoto, Heungjin Ryu, Keiko Mouri, Keiko Shimizu, Tetsuya Sakamaki, Takeshi Furuichi

**Affiliations:** 1 Primate Research Institute, Kyoto University, Kyoto, Japan; 2 School of Life Science, Ulsan National Institute of Science and Technology, Ulsan, Republic of Korea; 3 Faculty of Science, Okayama University of Science, Okayama, Japan; 4 Antwerp Zoo Foundation, Royal Zoological Society of Antwerp, Antwerp, Belgium

## Correction to: Primates (2022) 63:109–121 10.1007/s10329-021-00968-w

The article “Physical, behavioral, and hormonal changes in the resumption of sexual receptivity during postpartum infertility in female bonobos at Wamba”, written by Chie Hashimoto, Heungjin Ryu, Keiko Mouri, Keiko Shimizu, Tetsuya Sakamaki, Takeshi Furuichi, was originally published Online First without Open Access. After publication in volume 63, issue 2, page 109–121 the author decided to opt for Open Choice and to make the article an Open Access publication. Therefore, the copyright of the article has been changed to © The Author(s) 2022 and the article is forthwith distributed under the terms of the Creative Commons Attribution 4.0 International License, which permits use, sharing, adaptation, distribution and reproduction in any medium or format, as long as you give appropriate credit to the original author(s) and the source, provide a link to the Creative Commons licence, and indicate if changes were made. The images or other third party material in this article are included in the article’s Creative Commons licence, unless indicated otherwise in a credit line to the material. If material is not included in the article’s Creative Commons licence and your intended use is not permitted by statutory regulation or exceeds the permitted use, you will need to obtain permission directly from the copyright holder. To view a copy of this licence, visit http://creativecommons.org/licenses/by/4.0/.

