# Supplementary material for: Physical, behavioral, and hormonal changes in the resumption of sexual receptivity during postpartum infertility in female bonobos at Wamba
Source: Primates. 2022 Feb 10;63(2):109–21. doi: 10.1007/s10329-021-00968-w (PMC9352606; doi:10.1007/s10329-021-00968-w)
Supplement: Supplementary file 1 — Supplementary file1 (DOCX 655 KB) [file 10329_2021_968_MOESM1_ESM.docx]

Online Resource 1. Presence of adult females and males in a party that was observed on each day in 2015. There were no observations on the shaded days.

Online Resource 2. Changes in E_1_C and PdG concentrations during a) a successful conception by Otomi in August 2014, b) a successful conception by Nao in August 2014, and c) a successful conception by Nao in January 2014, after failing to maintain pregnancy.

Online Resource 3. A composite profile of E_1_C and PdG concentrations during non-conception cycles relative to the estimated day of ovulation. This figure is composed of fourteen cycles with estimated ovulation from the nine females shown in Table 1. Error bars indicate standard deviation. Day 0 is the day of ovulation.

Online Resource 4. Timing of ovulation relative to periods of maximal swelling (MS) in 14 cycles, for which day of ovulation was estimated.

Blue cells indicate the days in which the females showed maximal swelling, and red cells with diagonal lines show the day of ovulation. e: ovulation cycle that resulted in early loss. p: ovulation cycle that resulted in successful pregnancy (parturition). Gray cells with crosses show the days on which these females were not observed.
